# Anti-tumor Effects of *Hedyotis diffusa* Willd on Cervical Cancer: Inhibition of Proliferation, Migration, and Induction of Apoptosis

**DOI:** 10.5812/ijpr-159390

**Published:** 2025-07-13

**Authors:** Siyu Yang, Lizhu Zeng, Caiyun Deng, Xianglian Tian, Wenkui Sun, Chengjie Ji, QingLian Zhang

**Affiliations:** 1 School of Laboratory Medicine, Chengdu Medical College, Chengdu, China; 2 Department of Nuclear Medicine, Affiliated Hospital of Southwest Medical University, Luzhou, China; 3 Department of Clinical Laboratory, Jianyang People's Hospital, Jianyang, China

**Keywords:** *Hedyotis diffusa* Willd, Anti-cancer Effect, Cervical Cancer, Apoptosis, Cell Cycle Arrest

## Abstract

**Background:**

Cervical cancer is among the most prevalent malignancies affecting women globally. This study examines the anti-tumor effects and mechanisms of *Hedyotis diffusa* Willd (HDW), a traditional Chinese medicinal herb, on cervical cancer.

**Methods:**

The effects of HDW on the proliferation and migration of SiHa and CaSki cells were evaluated through in vitro assays. Additionally, the anti-tumor activity of HDW was assessed in vivo using a xenograft mouse model. To further explore its potential mechanisms, the effects of HDW on apoptosis, the cell cycle, and the IL-17/NF-κB signaling pathway were analyzed.

**Results:**

In vitro, HDW dose-dependently reduced the proliferation, colony formation, and migration of SiHa and CaSki cervical cancer cells (P < 0.01). It induced apoptosis by modulating the expression of BCL2, BAX, caspase 3, and cleaved-caspase 3, and caused S-phase cell cycle arrest through suppression of CDK2 and cyclin A (P < 0.05 or P < 0.01). *Hedyotis diffusa* Willd also inhibited the IL-17/NF-κB signaling pathway by reducing IL-17A and phosphorylated NF-κB p65 expression (P < 0.05 or P < 0.01). In vivo, HDW significantly inhibited tumor growth in a xenograft mouse model (n = 5), with no signs of systemic toxicity, as demonstrated by stable body weight.

**Conclusions:**

These findings suggest that HDW exerts its anti-tumor effects by inducing apoptosis, causing cell cycle arrest, and inhibiting the IL-17/NF-κB signaling pathway, highlighting its potential as a promising therapeutic candidate for cervical cancer.

## 1. Background

Cervical cancer is a preventable malignancy affecting women; however, its incidence and mortality rates have been increasing in developing countries in recent years. Globally, it ranks as the fourth most common cancer among women, with an estimated 660,000 new cases and 340,000 deaths in 2022, surpassing the figures from 2020 and 2018 (1-3). The standard treatment for cervical cancer often involves concurrent radiation and chemotherapy, primarily using cisplatin (CDDP) (4). However, the severe side effects and resistance associated with CDDP and other first-line chemotherapeutics significantly reduce their effectiveness, resulting in low response rates and decreased overall survival (5). Therefore, there is an urgent need for the development of new anticancer drugs. In clinical practice, traditional Chinese medicine remains a widely favored alternative for treating human papillomavirus-related diseases due to its multi-target effects, safety profile, and affordability (6). *Hedyotis diffusa* Willd (HDW) is a renowned medicinal plant with diverse therapeutic properties, including anti-inflammatory, antioxidant, anti-fatigue, and immune-enhancing effects, as well as notable anticancer activity (7). Notably, HDW has exhibited potential in suppressing the progression of various cancers, such as liver cancer (8, 9), colorectal cancer (10), lung cancer (11), and others (12). However, its application in treating cervical cancer has been limited.

## 2. Objectives

The present study aimed to investigate the anti-tumor effects of HDW on cervical cancer, potentially offering a novel therapeutic approach for cervical cancer treatment.

## 3. Methods

### 3.1. Cell Culture and Treatment

Human cervical cancer cell lines SiHa and CaSki were sourced from Procell Life Science & Technology (Wuhan, China) and maintained under standard culture conditions. *Hedyotis diffusa* Willd extract was purchased from Shanghai Yuanye Bio-Technology Co., Ltd. (HDW, Lot No.: R02S11Y123184, 20:1) and is a water-soluble brown powder. For treating cells and animals, the HDW extract was dissolved in cell culture medium for in vitro experiments and in 0.9% normal saline for in vivo administration.

### 3.2. Cell Viability Assay

Cell viability was assessed using a CCK8 kit (Biosharp, Beijing, China). SiHa and CaSki cells (5 × 10^3^ cells/well) were grown in 96-well plates containing 100 µL of complete medium and maintained at 37°C in a 5% CO_2_ environment. After cell adhesion, the cells were treated with varying concentrations of HDW for 24 or 48 hours. Subsequently, CCK8 reagent was introduced to each well and allowed to incubate for 2 hours. Using a microplate reader, the optical density (OD) at 450 nm was measured, and the 50% inhibitory concentration (IC_50_) was calculated with GraphPad Prism 9 software.

### 3.3. Colony Formation Assay

For colony formation analysis, SiHa and CaSki cells were seeded in 6-well plates at a density of 1 × 10^3^ cells per well. After adhesion, the cells were exposed to various concentrations of HDW and incubated for 14 days. Formed colonies were fixed with 4% paraformaldehyde, stained with Giemsa (Solarbio, Beijing, China), and subsequently counted for analysis.

### 3.4. Wound Healing Assay

Cells were seeded in 6-well plates, and once a confluent monolayer was achieved, a sterile pipette tip was used to create a scratch. The cells were then treated with various concentrations of HDW and incubated for 48 hours. Cell migration was quantified by measuring the scratch width before and after treatment.

### 3.5. Cell Apoptosis and Cell Cycle Assays

SiHa and CaSki cells (2 × 10^5^ cells/well) were seeded in 6-well plates. After adhesion, the cells were exposed to varying concentrations of HDW for 48 hours, harvested, and processed for apoptosis or cell cycle analysis. Apoptosis was assessed using the Annexin V-FITC/Propidium Iodide Apoptosis Detection Kit (KeyGen BioTECH, Jiangsu, China). Cell cycle analysis was performed using a cell cycle reagent (Solarbio, Beijing, China). Samples were incubated at 37°C and analyzed using a flow cytometer (Novocyte).

### 3.6. Tumor Xenograft Mice Models

Five-week-old female BALB/c nude mice were sourced from GemPharmatech Co., Ltd. (China). To create tumor xenograft models, 8 × 10^6^ SiHa cells were subcutaneously injected into the right dorsal flank of each mouse. Mice were randomly divided into experimental and control groups (n = 5 per group). When tumor size reached 5 × 5 mm, mice in the experimental group received HDW treatment (600 mg/kg, administered by gavage), while the control group received 0.9% normal saline by gavage. Treatments were given once daily for 21 consecutive days. Tumor dimensions and body weight were assessed every three days. The formula used to calculate tumor volume was: Volume = length × (width)^2^/2. After the last dose and measurement, the mice were euthanized, and the tumors were removed, measured, and weighed.

### 3.7. Western Blotting

Cellular proteins were separated by SDS-PAGE and transferred onto PVDF membranes. The membranes were incubated overnight at 4°C with primary antibodies, washed, and incubated again with secondary antibodies for 2 hours. Protein bands were visualized using an imager. The primary antibodies utilized in this study included cyclin A (AF0142), BCL2 (AF6139), cleaved caspase-3 (AF7022), and caspase-3 (AF6311) from Affinity; p-NF-κB p65 (AF5878) from Beyotime; GAPDH (52902) from Signalway Antibody; and p-IκBα (82349-1-RR), CDK2 (10122-1-AP), BAX (50599-2-Ig), IL-17A (26163-1-AP), IκBα (66418-1-Ig), and NF-κB p65 (66535-1-Ig) from Proteintech.

### 3.8. Bioinformatics Analysis of the Molecular Mechanism Underlying *Hedyotis diffusa* Willd’s Inhibitory Effects on Cervical Cancer

The active components of HDW were identified using the Traditional Chinese Medicine Systems Pharmacology (TCMSP) database and analysis platform (13). Potential molecular targets for each active ingredient were predicted using the SwissTargetPrediction database with a probability of ≥ 0.9 (14). Microarray datasets of cervical cancer patients were obtained from the Gene Expression Omnibus (GEO) and The Cancer Genome Atlas (TCGA). Differentially expressed genes (DEGs) were identified using the "DESeq2" package in R 4.2.2, applying a threshold of log|(FC)| > 1.2 and P < 0.05. The overlap between the predicted targets of HDW’s active ingredients and the DEGs from cervical cancer datasets was obtained using the Venn diagram tool (15). These overlapping genes were considered potential target genes of HDW against cervical cancer. To elucidate the potential molecular mechanisms, enrichment analyses of the overlapping genes were conducted using Gene Ontology (GO) and Kyoto Encyclopedia of Genes and Genomes (KEGG) pathway tools. The Benjamini-Hochberg (BH) method was applied to adjust P-values, with an adjusted P-value (P.adjust) < 0.05 considered statistically significant.

### 3.9. High-Performance Liquid Chromatography-Mass Spectrometry

A total of 100 mg of HDW extract was accurately weighed and extracted with 1 mL of aqueous solution containing 2 µg mixed internal standards. The mixture was vortexed for 1 minute, pre-cooled at -40°C for 2 minutes, and then sonicated in an ice bath for 60 minutes. After centrifugation at 12,000 rpm for 10 minutes at 4°C, the supernatant was collected and diluted 10-fold with water containing 2 µg/mL mixed internal standards. Then, 200 µL of the diluted sample was transferred to liquid chromatography-mass spectrometry (LC-MS) vials with glass inserts for analysis. Chromatographic separation was performed using an ACQUITY UPLC I-Class (Waters, Milford, MA, USA) equipped with an ACQUITY UPLC HSS T3 column (100 mm × 2.1 mm, 1.8 µm, Waters). The column temperature was maintained at 45°C. The mobile phases consisted of 0.1% formic acid in water (phase A) and acetonitrile (phase B), with a gradient elution program detailed in Appendix 1 in Supplementary File. The flow rate was set to 0.35 mL/min, and the injection volume was 2 µL. Detection was performed using a photodiode array (PDA) detector over a wavelength range of 210 - 400 nm. Mass spectrometric detection was conducted on a Thermo Orbitrap Q Exactive HF high-resolution mass spectrometer equipped with a heated electrospray ionization (HESI) source, operating in data-dependent acquisition (DDA) mode. The mass spectrometry parameters are listed in Appendix 2 in Supplementary File. Raw data were processed using Progenesis QI v3.0 software (Nonlinear Dynamics, Newcastle, UK), including baseline correction, peak detection, integration, retention time alignment, and normalization. Compound identification was based on accurate mass, MS/MS fragmentation patterns, and isotope distribution, and matched against the LuMet-TCM database.

### 3.10. Determination of Total Flavonoid Content

The total flavonoid content of the HDW extract was determined using a colorimetric assay based on the aluminum nitrate colorimetric method, with rutin as the standard. A standard calibration curve was constructed using a series of rutin solutions at known concentrations. For sample preparation, 0.5 g of the dried HDW extract was weighed and ultrasonically extracted with 20 mL of 70% ethanol for 1 hour. The extract was centrifuged at 4000 rpm for 10 minutes, and 200 µL of the resulting supernatant was mixed with 4.8 mL of distilled water. Subsequently, 0.6 mL of 5% NaNO_2_ solution was added, and the mixture was allowed to stand for 6 minutes. Then, 0.6 mL of 10% Al(NO_3_)_3_ solution was added, followed by another 6-minute incubation. Afterward, 3 mL of 4% NaOH solution and 0.8 mL of distilled water were added to complete the color development. The absorbance was measured at 510 nm using a UV-Vis spectrophotometer. The total flavonoid content was calculated based on the standard curve and expressed as milligrams of rutin equivalent per gram of extract (mg/g).

### 3.11. Statistical Analysis 

Statistical analyses were performed using GraphPad Prism 9. The data are presented as the mean ± standard error of the mean (SEM). Statistical significance was determined using an unpaired Student’s *t*-test or one-way analysis of variance (ANOVA). All experiments were conducted in triplicate, with a P-value < 0.05 regarded as statistically significant.

## 4. Results

### 4.1. *Hedyotis diffusa* Willd Exhibits Cytotoxic and Anti-migratory Effects on Cervical Cancer Cells 

**Figure 1.**
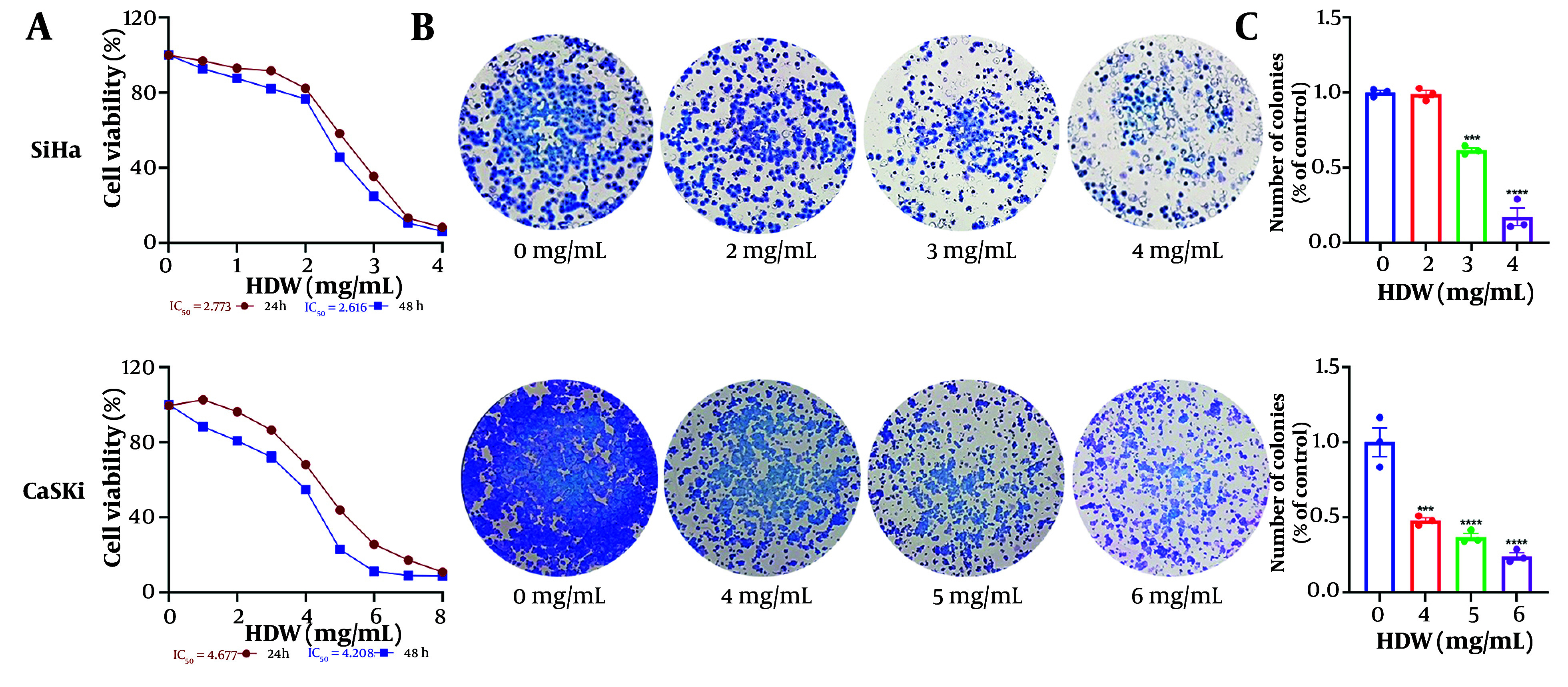
The effects of *Hedyotis diffusa* Willd (HDW) on cell survival and colony formation. A, the effects of HDW on cell survival, the horizontal axis is the concentration of HDW, and the vertical axis is cell viability. SiHa and CaSki cells were treated with various concentrations of HDW for 24 or 48 hours respectively, and its cell survival was estimated by CCK8 assays; B, the effects of HDW on colony formation, the dosage of 2, 3, and 4 mg/mL were used on SiHa cells (the upper) and dosage of 4, 5, and 6 mg/mL were used on CaSki cells (the lower) respectively, and the groups without HDW was set as the control; C, the histogram of colony formation assays. Statistical significance is indicated as follows: *** P < 0.001, and **** P < 0.0001.

**Figure 2.**
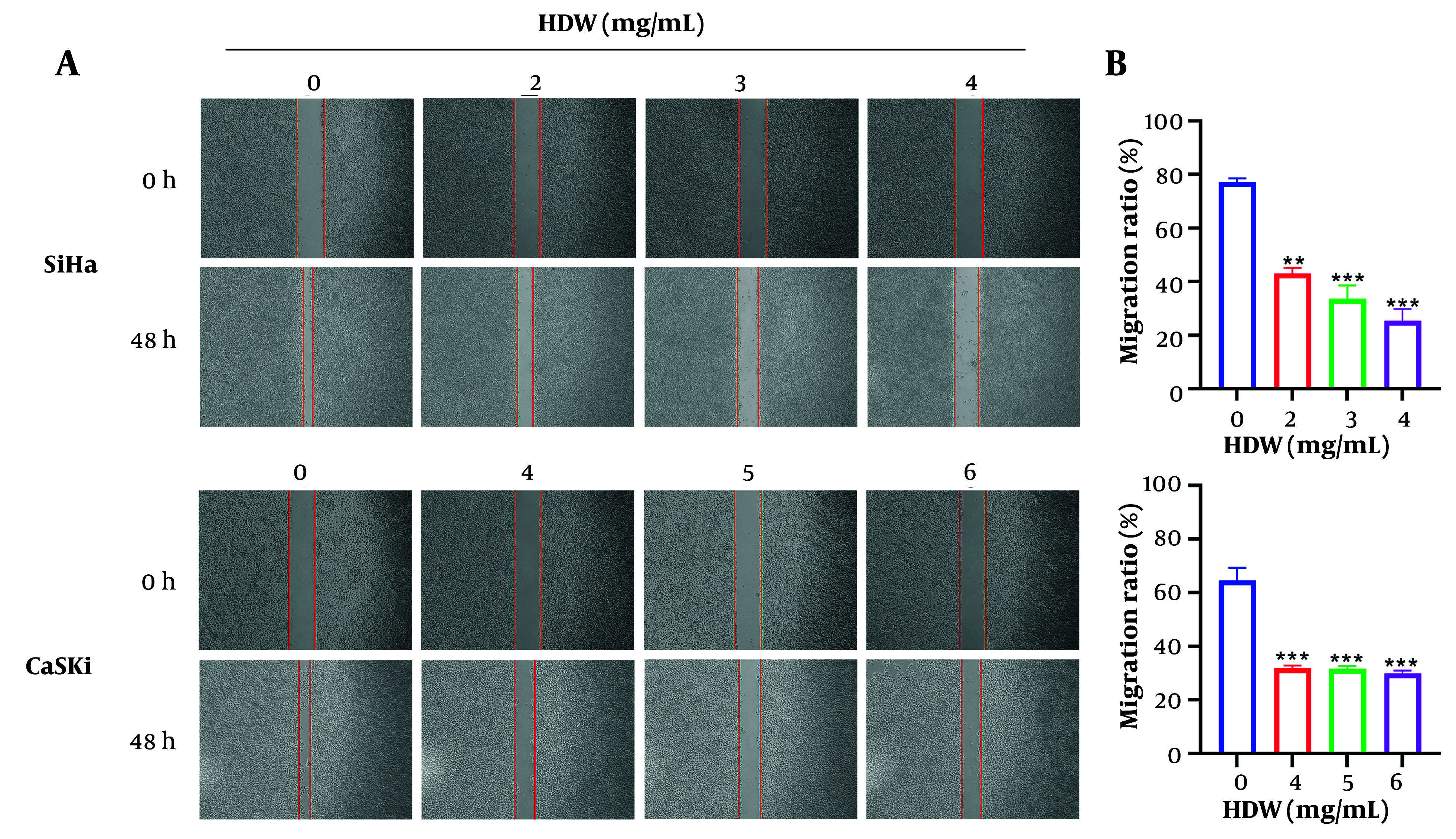
The effects of *Hedyotis diffusa* Willd (HDW) on cell migration. A, the images of scratches before and after using of HDW, the dosage of 2, 3, and 4 mg/mL were used on SiHa cells (the upper) and dosage of 4, 5, and 6 mg/mL were used on CaSki cells (the lower) respectively, and the groups without HDW was set as the control. The red vertical line is the edge location of the cell, and the width between the two vertical lines represents the scratch width; B, the histogram of cell migration assay. Statistical significance is indicated as follows: ** P < 0.01, and *** P < 0.001.

SiHa and CaSki cells treated with varying doses of HDW exhibited dose-dependent cytotoxicity, as determined by the CCK8 assay. The IC_50_ values for SiHa cells were 2.773 mg/mL at 24 hours and 2.616 mg/mL at 48 hours, while those for CaSki cells were 4.677 mg/mL and 4.208 mg/mL, respectively (Figure 1A). Additionally, HDW significantly inhibited colony formation in both cell lines, with higher concentrations of HDW leading to a marked reduction in colony numbers, consistent with the CCK8 assay results (Figure 1B and C). Furthermore, wound healing assays demonstrated that HDW impaired cell migration. The HDW-treated SiHa and CaSki cells exhibited significantly reduced scratch-healing ability compared with untreated controls, indicating a diminished capacity for lateral migration (Figure 2A and B).

### 4.2. *Hedyotis diffusa* Willd Suppresses Tumor Growth in Mice 

**Figure 3.**
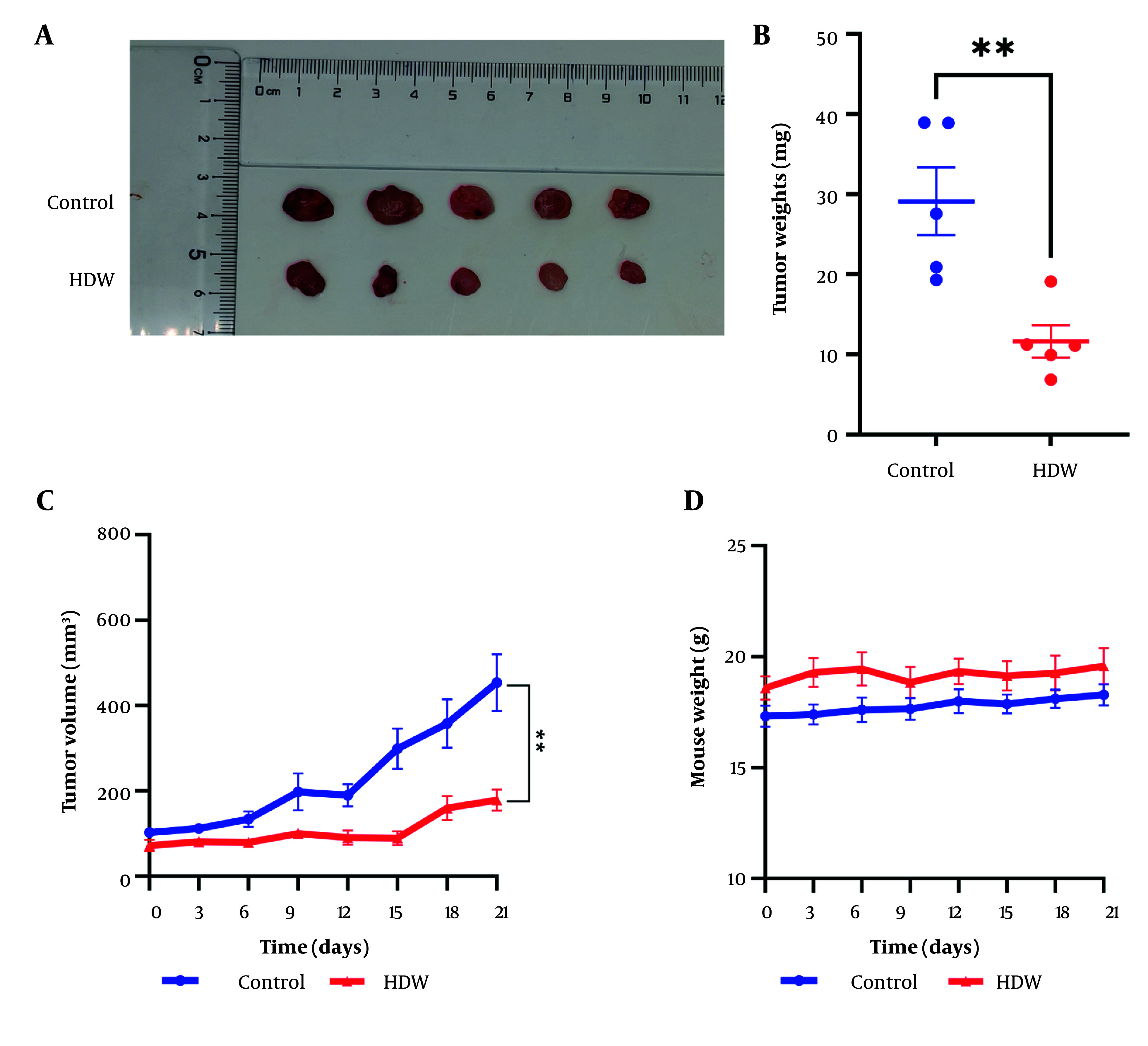
The effects of *Hedyotis diffusa*illd (HDW) on tumor growth in Xenograft mice. A, the tumors from HDW-treated mice (the lower) and control mice; B, tumor weights of the control group (the blue dots) compare with the HDW-treated group (the red dots); C, tumor growth curve of the HDW-treated group (the red) and the control group (the blue); D, weights of mice of the HDW-treated group (the red) and the control group (the blue). Statistical significance is indicated as follows: ** P < 0.01.

The anti-tumor efficacy of HDW was further evaluated using xenograft mice models. *Hedyotis diffusa* Willd treatment markedly suppressed tumor growth, resulting in significant reductions in tumor volume and weight compared with the control group (Figure 3A - C). Importantly, no significant decrease in body weight was observed in HDW-treated mice, suggesting minimal systemic toxicity (Figure 3D). These findings demonstrate that HDW effectively inhibits tumor growth in vivo and underscore its potential as a therapeutic agent for cervical cancer.

### 4.3. Potential Molecular Mechanism of *Hedyotis diffusa* Willd’s Inhibitory Effects on Cervical Cancer

**Figure 4.**
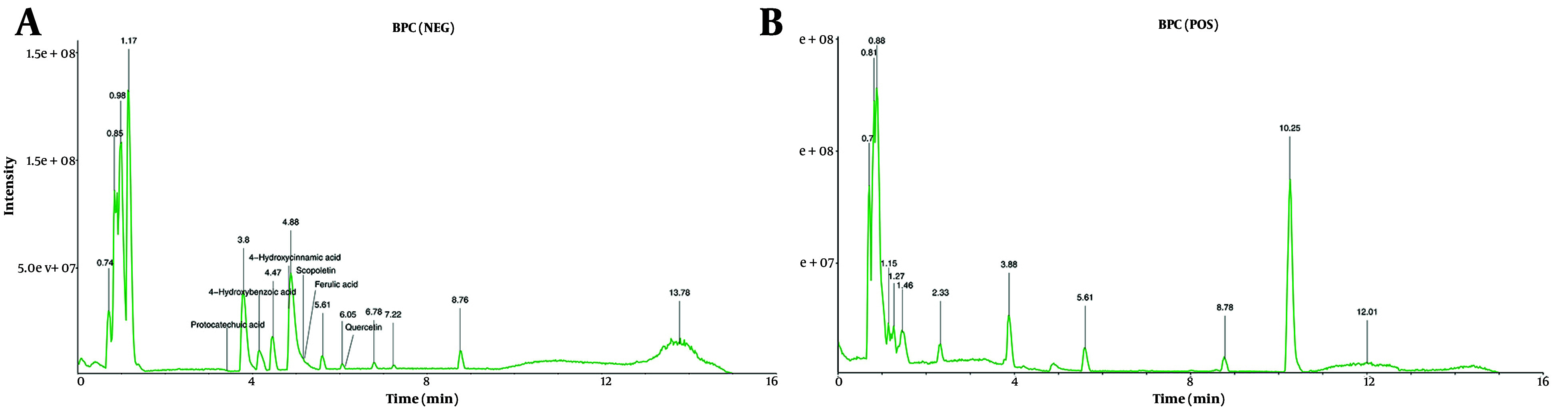
Base peak chromatograms (BPC) of the *Hedyotis diffusa* Willd (HDW) extract obtained by liquid chromatography-mass spectrometry (LC-MS) analysis. A, negative ionization mode; B, positive ionization mode. The active compounds of interest were annotated in the chromatograms.

**Figure 5.**
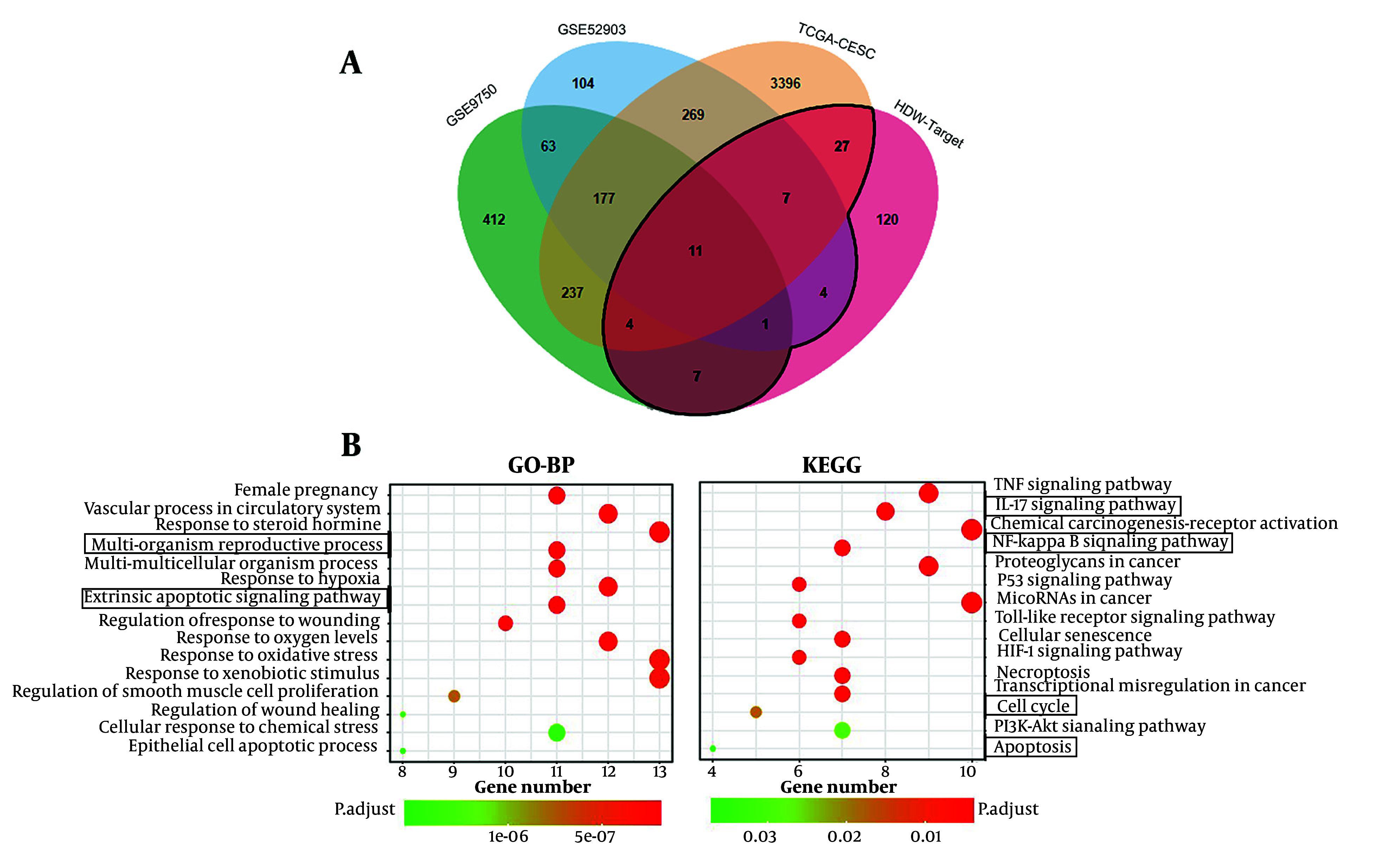
Enrichment analysis for the overlapping target genes of *Hedyotis diffusa* Willd (HDW) and cervical cancer. A, Venn diagram of the overlapping targets of HDW and CESC, the black line surrounds the intersecting genes; B, GO-BP enrichment analysis results showed that the multi-organism reproductive process and extrinsic apoptotic signaling pathway were significantly enriched. Kyoto Encyclopedia of Genes and Genomes (KEGG) enrichment analysis results showed that HDW may regulate IL-17, NF-κB, cell cycle and apoptosis signaling pathways in CESC.

Using the TCMSP and SwissTargetPrediction databases, 12 active compounds of HDW and their corresponding predicted targets were obtained (Table 1). After removing duplicates, a total of 181 unique target genes were identified (Appendix 3 in Supplementary File). To verify the presence of the predicted compounds in actual samples, LC-MS analysis was conducted. Both negative and positive ionization modes were analyzed (Figure 4A and B), but the target compounds were detected in the negative ionization mode. Among the 12 predicted compounds, protocatechuic acid, 4-hydroxybenzoic acid, 4-hydroxycinnamic acid, scopoletin, ferulic acid, and quercetin were successfully detected (Figure 4A). The remaining compounds were not detected, likely due to their concentrations being below the detection limit of LC-MS. Additionally, the total flavonoid content in the HDW extract was 36.10 ± 0.22 mg/g, indicating a relatively high abundance of pharmacologically active flavonoids (Appendices 4 and 5 in Supplementary File). To further investigate the potential anti-cervical cancer mechanisms of HDW, we analyzed GEO and TCGA datasets and identified 4,719 DEGs associated with cervical squamous cell carcinoma and endocervical adenocarcinoma (CESC). The intersection of these DEGs with the 181 predicted HDW targets yielded 61 overlapping genes, which may represent key therapeutic targets of HDW in the treatment of cervical cancer (Figure 5 Appendix 6 in Supplementary File). Enrichment analysis of these 61 overlapping genes revealed key biological processes that HDW may modulate, including IL-17, NF-κB, cell cycle regulation, and apoptosis pathways (Figure 5B). 

**Table 1.** Active Compounds of *Hedyotis diffusa* Willd and the Number of Their Predicted Targets

Pubchem CID	Molecular Name	Targets
**5280343**	Quercetin	121
**10514946**	2-methoxy-3-methyl-9,10-anthraquinon	30
**222284**	β-sitosterol	26
**5280794**	Stigmasterol	24
**5280863**	Kaempferol	17
**637542**	4-Hydroxycinnamic acid	13
**135**	4-Hydroxybenzoic acid	12
**72**	Protocatechuic acid	8
**445858**	Ferulic acid	8
**5280460**	Scopoletin	3
**5281330**	Poriferasterol	2
**11869658**	(4aS,6aR,6aS,6bR,8aR,10R,12aR,14bS)-10-hydroxy-2,2,6a,6b,9,9,12a-heptamethyl-1,3,4,5,6,6a,7,8,8a,10,11,12,13,14b-tetradecahydropicene-4a-carboxylic acid	1

### 4.4. *Hedyotis diffusa* Willd Induces Cell Apoptosis

**Figure 6.**
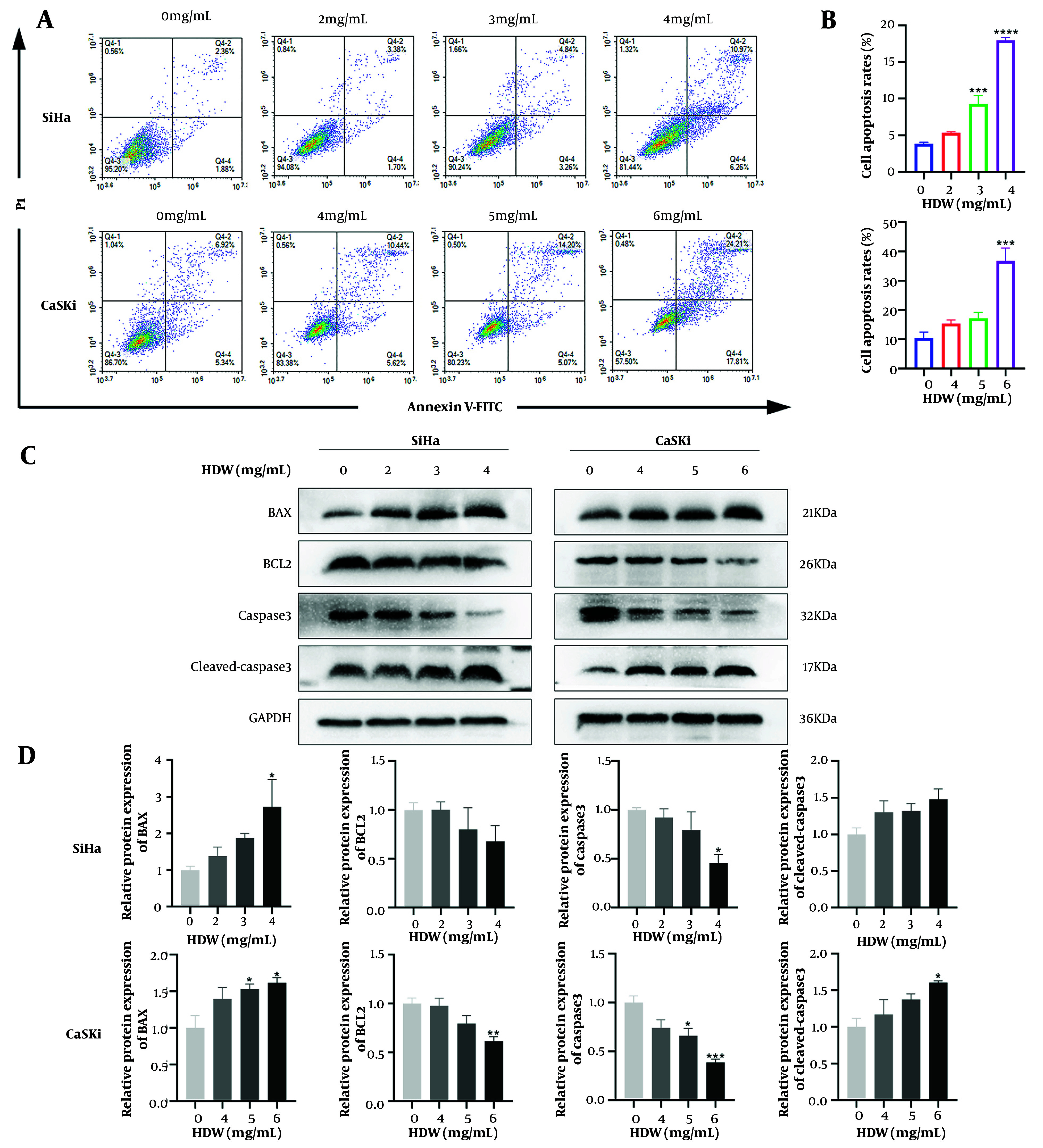
*Hedyotis diffusa* Willd (HDW) induces cell apoptosis. A, representative images of cell apoptosis after 48 hours of HDW treatment. SiHa cells were treated with HDW at concentrations of 2, 3, and 4 mg/mL (the upper), while CaSki cells were treated with 4, 5, and 6 mg/mL (the lower). Untreated cells served as the control group; B, quantitative histogram showing cell apoptosis rates across treatment groups; C, Western blot analysis of key apoptosis-related proteins; D, quantitative histogram of protein expression levels from Western blot analysis. (Abbreviations: PI, propidium iodide; BAX, Bcl-2-associated X protein; BCL2, B-cell lymphoma 2; caspase 3, cysteine-aspartic acid protease 3; cleaved-caspase 3, cleaved cysteine-aspartic acid protease 3; GAPDH, glyceraldehyde-3-phosphate dehydrogenase. Statistical significance is indicated as follows: * P < 0.05, ** P < 0.01, *** P < 0.001, and **** P < 0.0001.)

Flow cytometry analysis (Figure 6A and B) revealed that cell apoptosis rates increased in a dose-dependent manner with higher HDW concentrations. Furthermore, as the HDW dose increased, the expression levels of BCL2 and caspase 3 decreased. Conversely, the expression levels of BAX and cleaved-caspase 3 increased (Figure 6C and D).

### 4.5. *Hedyotis diffusa* Willd Blocks the Cell Cycle in the S Phase

**Figure 7.**
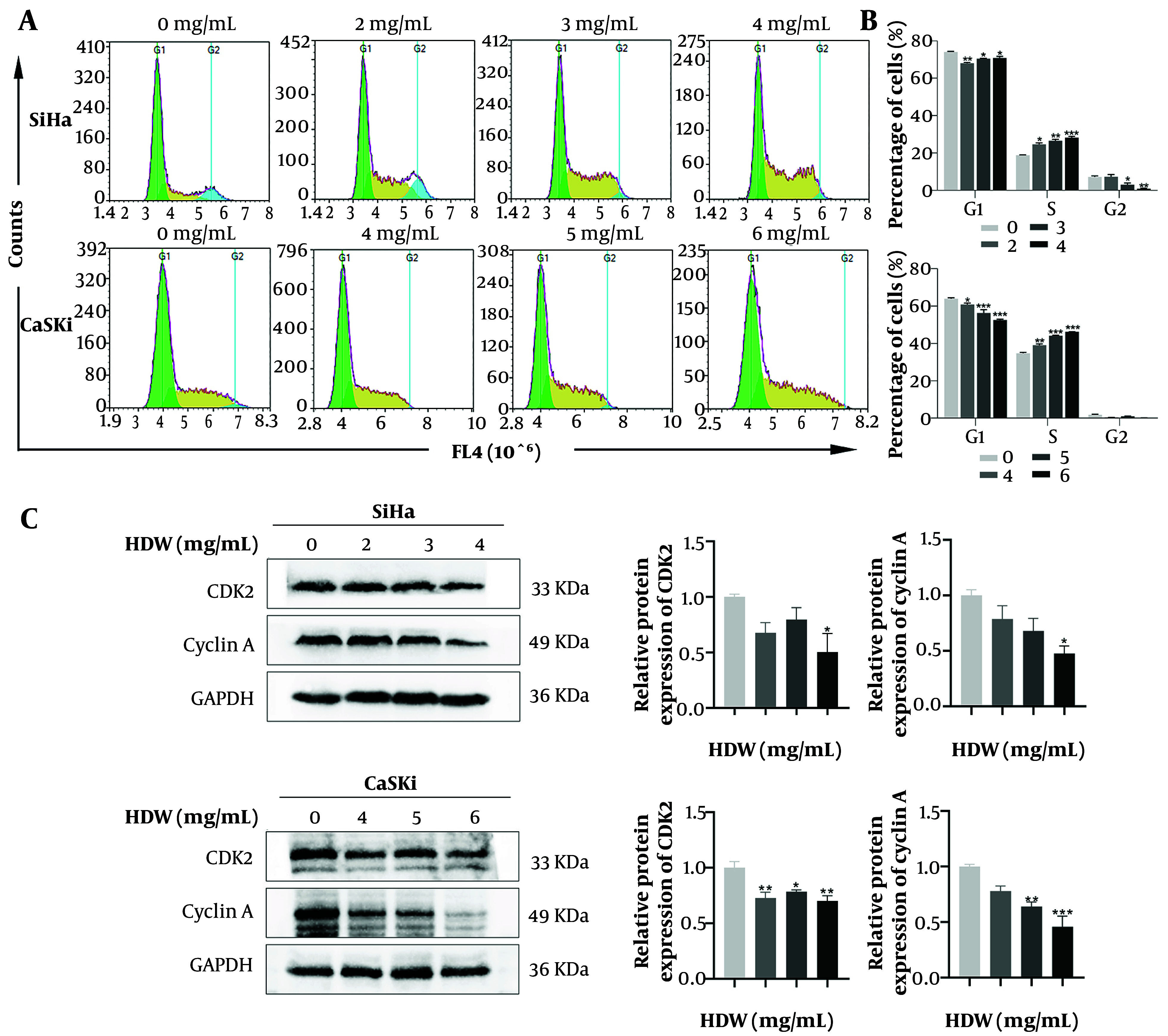
Effects of *Hedyotis diffusa* Willd (HDW) on the cell cycle. A, representative images showing cell cycle distribution after 48 hours of HDW treatment. Green represents the G1 phase, yellow represents the S phase, and blue represents the G2 phase; B, quantitative histogram of cell distribution across different cell cycle phases; C, Western blot analysis of key cell cycle regulatory proteins with quantitative histograms. (Abbreviations: CDK2, cyclin-dependent kinase 2; GAPDH, glyceraldehyde-3-phosphate dehydrogenase. Statistical significance is indicated as follows: * P < 0.05, ** P < 0.01, and *** P < 0.001.)

Flow cytometry analysis of the cell cycle revealed a progressive increase in the proportion of cells in the S phase as HDW concentrations rose (Figure 7A and B). Moreover, the expression levels of CDK2 and cyclin A, critical regulators of the S phase, decreased in parallel with the escalating HDW concentration (Figure 7C). 

### 4.6. *Hedyotis diffusa* Willd Inhibits the IL-17/NF-κB Signaling Pathways

**Figure 8.**
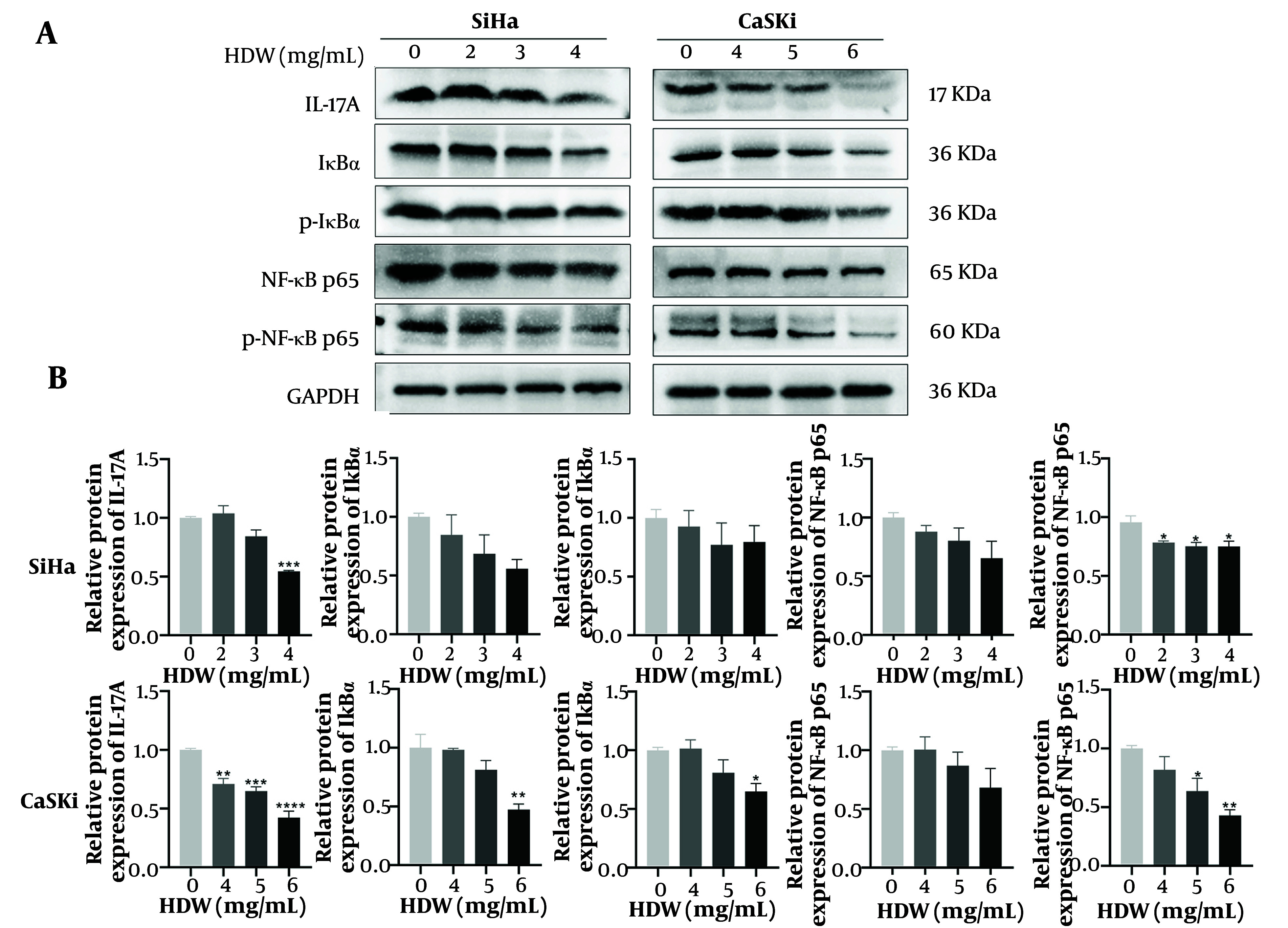
Effects of *Hedyotis diffusa* Willd (HDW) on the IL-17/NF-κB signaling pathway. A, western blot analysis of key proteins involved in the IL-17/NF-κB pathway; B, quantitative histogram of protein expression levels from Western blot analysis. (Abbreviations: IL-17A, interleukin 17A; IκBα, inhibitor of kappa B alpha; p-IκBα, phosphorylated Inhibitor of kappa B alpha; NF-κB p65, nuclear factor kappa B p65; p-NF-κB p65, phosphorylated nuclear factor kappa B p65, GAPDH, glyceraldehyde-3-phosphate dehydrogenase. Statistical significance is indicated as follows: * P < 0.05, ** P < 0.01, *** P < 0.001, and **** P < 0.0001.)

Western blot analysis revealed that HDW treatment significantly altered the expression and phosphorylation levels of key regulatory proteins in the IL-17 and NF-κB signaling pathways. Specifically, the expression of IL-17A and phosphorylated NF-κB p65 (p-NF-κB p65) was markedly reduced following HDW treatment (Figure 8A and B). These results indicate that HDW may effectively suppress the IL-17 and NF-κB signaling pathways CESC cells.

## 5. Discussion

This study provides the first evidence that HDW extract inhibits cell proliferation and migration, induces apoptosis, and causes cell cycle arrest in CESC cells, consistent with its reported effects on other cancer cells, such as breast, colorectal, and hepatocellular carcinoma (HCC). In breast cancer, HDW exerts its anti-cancer effects by activating the tumor suppressor p53 through the regulation of the ERα/Sp1 pathway (16). In colorectal cancer, HDW inhibits IL-6-induced phosphorylation of the STAT3 pathway and promotes apoptosis (17). In HCC, HDW inhibits cell proliferation and invasion by modulating the AKT/mTOR signaling pathway (9). *Hedyotis diffusa* Willd has been shown to trigger apoptosis in HCC via the JNK/Nur77 pathway, promoting the upregulation of BAX, cleaved-caspase 3, and cytochrome C, while downregulating BCL2 (18). Similarly, we find that HDW inhibited the expression levels of BCL2 and caspase-3, while promoting the expression levels of BAX and cleaved-caspase 3 in cervical cancer cells. These results suggest that HDW possibly promotes cell apoptosis by regulating the expression of these key proteins. BCL2 is one of the most important anti-apoptotic proteins that can prevent apoptosis by inhibiting the release of cytochrome C from the mitochondria into the cytoplasm, thus preventing cytochrome C from activating caspase proteases and inhibiting the apoptotic process. The BAX, on the other hand, is a pro-apoptotic protein that promotes the release of cytochrome C, triggering the apoptosis cascade. By modulating the balance between BCL2 and BAX, the apoptosis process can be regulated (19, 20). Our results show that HDW reduced the BCL2/BAX ratio, leading to the activation of caspase proteases, which convert caspase-3 into cleaved-caspase 3, the activated form of caspase-3, thus promoting apoptosis.

In HCC cells, HDW has been reported to downregulate CDK2 and E2F1 (21). E2F1, a transcription factor driving the G1/S transition, and CDK2, which also plays a role in regulating this transition, are downregulated in HCC cells, leading to G0/G1 phase arrest (22-24). Our study shows that HDW downregulates the expression of cyclin A and CDK2 proteins, which are key regulators of the G1 to S phase transition and promote DNA replication. By downregulating cyclin A and CDK2, HDW prevents cells from entering the S phase, leading to S-phase arrest in cervical cancer cells. However, the specific mechanism of HDW-induced cell cycle arrest remains unclear and requires further investigation to explore this process in depth.

Previous studies have reported that HDW significantly suppressed LPS-induced NF-κB activation and reduced the phosphorylation of MAPK signaling molecules, leading to the decreased expression of pro-inflammatory cytokines such as IL-6, TNF-α, IL-1β, and iNOS, further contributing to its anti-inflammatory effects (25). Consistent with these findings, we find that HDW reduced the expression levels of IL-17A and p-NF-κB p65, key proteins involved in the IL-17 and NF-κB signaling pathways, suggesting that HDW may exert its anti-cervical cancer effects through the suppression of these pathways.

Furthermore, to ensure the reliability of these biological effects, it is important to consider the quality and consistency of the HDW extract used in the study. The extract was prepared by a commercial supplier using standardized procedures, including botanical authentication and water extraction, which help ensure batch-to-batch consistency and chemical stability. Previous studies using extracts from the same supplier have shown reproducible anti-cancer activity, supporting the notion that the observed therapeutic effects are likely due to the synergistic actions of multiple components rather than a single active ingredient (9, 26-28). Nevertheless, natural product-based extracts are inherently subject to batch variability due to factors such as biological diversity, environmental conditions, and subtle differences in extraction processes (7, 29-31). These factors may influence the reproducibility and interpretation of experimental results and constitute a limitation of this study. Future research will focus on refining quality control standards and developing more robust analytical methods to enhance the reproducibility and reliability of such natural product research.

### 5.1. Conclusions

Our study is the first to evaluate the antitumor activity of HDW in CESC. We found that HDW inhibited the proliferation, colony formation, and migration of cervical cancer SiHa and CaSki cells. Additionally, HDW suppressed the growth of xenograft tumors. Further analysis showed that HDW inhibited the expression of phosphorylated NF-κB p65, IL-17A, CDK2, and cyclin A, and reduced the BCL2/BAX expression ratio. These results suggest that HDW exerts its antitumor effects possibly by inhibiting the IL-17/NF-κB pathway and disrupting cell cycle progression. Therefore, HDW demonstrates potential as a candidate for anti-tumor agent development in cervical cancer research.

ijpr-24-1-159390-s001.pdf

## Data Availability

The dataset is available from the corresponding author upon reasonable request. The data are not publicly available because they are essential for the author’s ongoing academic work and thesis completion.
