# Supplementary material for: Anti-tumor Effects of Hedyotis diffusa Willd on Cervical Cancer: Inhibition of Proliferation, Migration, and Induction of Apoptosis
Source: Iran J Pharm Res. 2025 Jul 13;24(1):e159390. doi: 10.5812/ijpr-159390 (PMC12524121; doi:10.5812/ijpr-159390)
Supplement: ijpr-24-1-159390-s001.pdf [file ijpr-24-1-159390-s001.pdf]

Appendix 1. Gradient Elution Program

Time (min)	A%	B%
0	95	5
2	95	5
4	70	30
8	50	50
10	20	80
14	0	100
15	0	100
15.1	95	5
16	95	5

Appendix 2. The Mass Spectrometry Parameters

Parameters	NEG	POS
Spray Voltage (V)	3800	-3200
Capillary Temperature (°C)	320	320
Aux Gas Heater Temperature(°C)	350	350
Sheath Gas Flow Rate (Arb)	35	35
Aux Gas Flow Rate (Arb)	8	8
S-lens RF level	50	50
Mass Range (m/z)	100-1500	100-1500
Full Ms Resolution	60000	60000
MS/MS Resolution	15000	15000
NCE/stepped NCE	10，20，40	10，20，40

Appendix 3. The 181 potential targets of HDW

Targets
PTGS1, AR, PPARG, PTGS2, HSP90, NCOA2, TOP2, KCNH2, SCN5A, ADRB2, MMP3, F7, RXRA, ACHE, RELA, EGFR, AKT1, VEGFA, CCND1, BCL2, BCL2L1, CDKN1A, BAX, CASP9, PLAU, MMP2, MMP9, MAPK1, IL10, EGF, RB1, TNF, IL6, AHSA1, CASP3, TP53, ELK1, NFKBIA, POR, ODC1, XDH, CASP8, TOP1, RAF1, PRKCA, MMP1, HIF1A, STAT1, RUNX1T1, ERBB2, ACACA, HMOX1, CYP3A4, CAV1, MYC, F3, GJA1, CYP1A1, ICAM1, IL1B, CCL2, SELE, VCAM1, PTGER3, PRKCB, BIRC5, DUOX2, NOS3, HSPB1, IL2, NR1I2, CYP1B1, CCNB1, PLAT, THBD, SERPINE1, IFNG, IL1A, MPO, TOP2A, NCF1, HAS2, GSTP1, NFE2L2, AHR, PSMD3, SLC2A4, CXCL11, CXCL2,

DCAF5, NR1I3, CHEK2, INSR, CLDN4, PPARA, PPARD, HSF1, CRP, CXCL10, CHUK, SPP1, RUNX2, RASSF1, E2F1, E2F2, ACP3, CTSD, IGFBP3, IGF2, CD40LG, IRF1, ERBB3, PON1, DIO1, PCOLCE, NPEPPS, HK2, NKX3-1, RASA1, GSTM1, GSTM2, DRD1, CHRM3, CHRM1, ESR1, CHRM5, CHRM4, OPRD1, PDE3A, HRH1, HTR2A, SLC6A2, ADRA1A, CHRM2, ADRA1B, SLC6A3, ADRA1D, SLC6A4, OPRM1, GABRA1, PIK3CG, CHRNA7, PKIA, PGR, CHRNA2, TGFB1, MAP2, NR3C2, ADH1C, NCOA1, ADRA2A, LTA4H, MOB, MOA, CTRB1, ADRB1, NOX4, AKR1B1, TYR, FLT3, CA2, ALOX5, CA7, HSD17B2, ABCC1, HSD17B1, CA12, ESRRA, ABCB1, ABCG2, CA1, CA3, CA4, CA9, CA5A, CA5B, CA6, CA14, ESR2, CA13, PTPN1

Appendix 4. Rutin standard curve for total flavonoid content determination by the aluminum nitrate colorimetric method

Rutin (mg/mL)	0	0.012	0.024	0.036	0.048	0.06	0.072
Absorbance	0.0000	0.0407	0.0990	0.1557	0.1983	0.2663	0.3237
Standard curve	y = 4.5288x - 0.0082 (R2 = 0.9969)						
	x: Rutin concentration (mg/mL); y: Absorbance						

Appendix 5. Quantitative analysis of total flavonoid content in HDW extract

Item	Group 1	Group 2	Group 3
Absorbance	0.206	0.205	0.204
Measured Concentration (mg/mL)	0.03634	0.03612	0.03590
Flavonoid Content (mg RE/g extract) (Amount / Weight)	36.34	36.12	35.90
Mean ± SD (mg RE/g extract, n=3)	36.12 ± 0.22		

Appendix 6. The 61 potential therapeutic targets of HDW against CESC

Targets
E2F2, PTGER3, ERBB3, BIRC5, E2F1, CAV1, PCOLCE, IFNG, IL10, DUOX2, PRKCA, HK2, NOS3, INSR, BCL2, SLC2A4, CHEK2, TNF, RUNX1T1, PLAU, MMP2, CYP1B1, IL1A, SPP1, MMP1, TOP2A, MMP9, AR, CCNB1, MMP3, CXCL11, CXCL10, ACP3, STAT1, CLDN4, IRF1, ICAM1, PLAT, IL1B, GJA1, IGFBP3, BAX, CCND1, NCF1, VCAM1, CXCL2, ADRB1, ADRA1A, ADRA2A, PGR, ADH1C, ABCB1, HSD17B1, ESRRA, ABCG2, CA3, CA4, CA9, CA2, ADRA1D, ESR1
